# Analysis and Identification of QTL for Resistance to *Sclerotinia sclerotiorum* in Pea (*Pisum sativum* L.)

**DOI:** 10.3389/fgene.2020.587968

**Published:** 2020-11-19

**Authors:** Rahil Ashtari Mahini, Ajay Kumar, Elias M. Elias, Jason D. Fiedler, Lyndon D. Porter, Kevin E. McPhee

**Affiliations:** ^1^Plant Science Department, North Dakota State University, Fargo, ND, United States; ^2^USDA-ARS, Cereal Crops Research Unit, Edward T. Schafer Agricultural Research Center, Fargo, ND, United States; ^3^USDA-ARS, Grain Legume Genetics and Physiology Research Unit, Prosser, WA, United States; ^4^Plant Sciences and Plant Pathology Department, Montana State University, Bozeman, MT, United States

**Keywords:** *Sclerotinia sclerotiorum*, *Pisum sativum*, QTL mapping, lesion expansion inhibition, nodal transmission inhibition, genotyping by sequencing, white mold

## Abstract

White mold caused by *Sclerotinia sclerotiorum* is an important constraint to field pea (*Pisum sativum* L.) production worldwide. To transfer white mold resistance into an adapted background, and study the genetics of the disease, two recombinant inbred line (RIL) populations (PRIL17 and PRIL19) were developed by crossing two partially resistant plant introductions with two susceptible pea cultivars. PRIL17 (Lifter × PI240515), and PRIL19 (PI169603 × Medora) were evaluated for resistance to white mold by measuring lesion expansion inhibition (LEI) and nodal transmission inhibition (NTI) at 3, 7, and 14 days post inoculation (dpi) under controlled environmental conditions. Lesion expansion inhibition percentage (LEIP), survival rate (SR), and area under disease progress curves (AUDPC) were also calculated accordingly. Because of a positive correlation between LEI and NTI with height, short and long internode individuals of each population were analyzed separately to avoid any confounding effect of height to pathogen response. A total of 22 short genotypes demonstrated partial resistance based on at least two Porter's resistance criteria. Only two pea genotypes with partial resistance to white mold (PRIL19-18 and PRIL19-124) had both semi-leafless (afila) and short internode traits. Both the RIL populations were genotyped using genotyping by sequencing (GBS). For PRIL17 and PRIL19, genetic maps were constructed from a total of 1,967 and 1,196 single nucleotide polymorphism (SNP) and spanned over 1,494 cM and 1,415 cM representing seven and nine linkage groups, respectively. A consensus map constructed using data from both populations, had 1,486 unique SNPs over 2,461 cM belonging to seven linkage groups. Inclusive composite interval mapping (ICIM) identified thirteen quantitative trait loci (QTL) associated with white mold resistance traits in both populations. Three of them were co-located with height genes (a morphological trait that reduces infection risk and acts as disease avoidance) and the other ten QTL were associated with two forms of physiological resistance (seven for LEI and three for NTI) with LOD and r^2^ ranging from 3.0 to 28.5 and 5.1 to 64.3, respectively. The development of resistance lines, genetic dissection and identification of markers associated will help accelerate breeding efforts for white mold resistance using molecular breeding approaches.

## Introduction

White mold caused by *Sclerotinia sclerotiorum* (Lib.) de Bary, causes significant yield losses in most dicotyledonous crops (Bolton et al., 2006; Shahoveisi and del Rio Mendoza, 2020). This hemi-biotrophic fungus has a host range of more than 400 plant species. White mold preferably develops in cool, cloudy, wet, and humid weather during flowering (Mueller et al., 2014). Narrow row spacing, and an early canopy closure typically by long-vine plants creates ideal conditions for mycelium germination and development of the disease (Kraft and Pfleger, 2001). The pathogen infects the stem, leaf or pod tissue of plants and produces water-soaked lesion with white cottony mass of mycelium on the surface (Mueller et al., 2014).

Annual losses from *S. sclerotiorum* in pulse crops was estimated to be as high as $12 million in the United States (USDA-ARS, 2016). White mold is a significant barrier to field pea production, not only in the Northwest and Midwest areas of the USA, but worldwide (Porter et al., 2009). The most economical and environment-friendly option for management of white mold is to develop resistant field pea varieties (Fernando et al., 2004; Jain et al., 2012; Peltier et al., 2012). However, breeding for white mold resistant varieties is difficult in most of the crops, because of its polygenic inheritance (Porter et al., 2009; Davar et al., 2013). Porter et al. (2009) stated that the expression of the partial quantitative white mold resistance in pea might be in two forms, lesion expansion inhibition (LEI) and nodal transmission inhibition (NTI These two forms are a measure of rate of lesion progression and ability of pathogen to spread in the stem through the nodes, respectively (Porter et al., 2009). Some morphological traits such as stem thickness, short internodes and leaf morphology (semi-leafless), can also influence white mold resistance (Porter et al., 2009). Plant height in soybean (Boland and Hall, 1986) and sunflower (Bazzalo et al., 1991) had shown positive correlation with resistance, resulting in disease escape. Limited attempts to screen pea germplasm for white mold resistance (Blanchette and Auld, 1978; Porter et al., 2009; Tashtemirov, 2012) identified only moderate levels of disease resistance. In this background, genetic dissection of the available disease resistance would be valuable to pyramid resistant genes from diverse sources.

Quantitative trait loci (QTL) mapping identification for *S. sclerotiorum* resistance has been reported in many crops including sunflower (Micic et al., 2005), soybean (Bastien et al., 2014; Iquira et al., 2015), bean (Kolkman and Kelly, 2003; Ender and Kelly, 2005; Miklas, 2007), rapeseed (*Brassica napus*) (Wu et al., 2013), and *Brassica oleracea* (Mei et al., 2013). However, only limited research on *S. sclerotiorum* resistance has been reported in pea (Tashtemirov, 2012). The only reported QTL mapping study for resistance to white mold in pea was conducted on F_2−_derived F_3_ family lines from a cross between Lifter and PI240515 (Tashtemirov, 2012). The recent availability of full genome sequence of the field pea (Kreplak et al., 2019) has contributed to a surge in identification of trait-associated markers and candidate genes for a number of agronomic, seed morphology and seed quality traits (Gali et al., 2019; Dissanayaka et al., 2020), and frost (Beji et al., 2020) and heat tolerance (Tafesse et al., 2020). Similarly, we expect that these genomic resources will also contribute to fine mapping of disease resistance QTLs including those associated with white mold resistance and a better understanding of the underlying genetics. Therefore, this study was conducted to (1) develop a reliable screening method for white mold resistance, (2) identify white mold resistant individuals in two RIL populations, and (3) detect QTL associated with white mold resistance in two mapping populations for marker-assisted selection in breeding programs.

## Materials and Methods

### Plant Materials

Two mapping populations of pea were used for this study: (i) PRIL17 comprised of 192 F_7_ recombinant inbred lines (RILs) derived from the cross of Lifter/ PI240515 and (ii) PRIL19 comprised of 324 F_7_ RILs developed from the cross PI169603/Medora. Both populations were developed using single seed decent. Lifter and Medora are susceptible cultivars, while PI240515 and PI169603 have partial resistance to white mold (Porter et al., 2009). Lifter, developed by the USDA-ARS (Grain Legume Genetics Physiology Research unit located in Pullman, WA), has short internodes with normal leaf type, white flowers and green seed (McPhee and Muehlbauer, 2002). Medora, which was developed for Midwest production, has a short internode with afila leaf type, white flowers and smooth green seed color (Grain Legume Genetics and Physiology Research Unit, 2007). The two partially resistant plant introductions were selected based on a previous study (Porter et al., 2009). PI240515 (originated from India) and PI169603 (originated from Turkey) have long internodes, normal leaf morphology, white flowers and yellow cotyledon color (https://www.ars-grin.gov/).

### Inoculum Preparation

Sclerotia of *Sclerotinia. sclerotiorum* isolate Sc102 were obtained from pea cultivar named “Snake” in 2003 by Dr. Lyndon Porter in Quincy, WA. Sclerotia were maintained at 4°C until used. To break sclerotia dormancy, they were placed in a 10% bleach solution for 20 min, followed by 3 rinses with sterile distilled water. The rinsed sclerotia were surface sterilized in 95% ethanol for one minute, briefly flamed and cultured on sterile composite agar in dark at 21–23°C for three days until the mycelium colonized half the surface of a 15 mm diameter petri dish (Khan et al., 2020). The composite agar consisted of 18.5 g Difco TM potato dextrose agar and 8.75 g Difco TM oatmeal agar in 0.5 L distilled water and autoclaved at 121 C for 20 min (Tashtemirov, 2012). Actively growing mycelium from the leading edge of the colony was used for inoculation of mapping populations.

### Phenotyping and Greenhouse Evaluation

Individual RILs grown in greenhouse were inoculated with *S. sclerotiorum* 14 days after sowing, using the jumbo agar plug technique (Porter et al., 2009). Plants were grown under natural sunlight supplemented with 600-Watt high pressure sodium lamps (P. L Light Systems, Inc., Beamsville, Ontario, Canada) in the greenhouse to maintain a 16:8 h photoperiod and temperatures from 20 to 25°C during the day and 20°C at night. The experiment was arranged in a randomized complete block design (RCBD) with 4 replicates and repeated three times. A total of 186 genotypes from PRIL17 and 140 individuals from PRIL19, in addition to 5 checks, were evaluated. The populations were divided indifferent experiment sets for logistic reasons (lack of space and labor).

The test plants were inoculated by placing 3 mm of actively growing mycelial plug at the 4th node using a jumbo pulpdent amalgam carrier and the media was pressed into the leaf axis. After inoculation, plants were transferred to a mist chamber with 100% humidity and 19 to 21°C temperature for three days in the dark. White mold lesions were measured 3 days post inoculation (dpi) with a digital caliper (mm). Plants were then transferred to a mist room for another 11 days with 80% humidity and 14:10 day: night photoperiod with supplemental lighting using 400-Watt high pressure sodium lamps (P. L. Light Systems, Inc., Beamsville, Ontario, Canada).

Lesion expansion inhibition (LEI) was measured by recording lesion size in millimeters at 3, 7, and 14 dpi where a smaller value indicated greater LEI. Nodal transmission inhibition (NTI) were measured at 7 and 14 dpi. NTI was scored on a scale of 0-4 based on lesion movement from the 4th node down as described in Porter et al. (2009). A larger value indicated greater NTI. Lesion expansion inhibition percentage (LEIP) was recorded 14 dpi by measuring lesion size (mm) and then dividing by plant height. Survival rate (SR) was calculated 14 dpi by measuring the uninfected portions of the main stems and dividing by plant height ([height at 14 dpi–lesion size at 14 dpi/ height in 14 dpi] ×100).

Means for LEI, NTI, LEIP and SR were calculated using ANOVA (analysis of variance) and PROC MIXED procedures in SAS Enterprise Guide 7.1 (SAS Institute Inc. USA). In the statistical analysis, genotypes were considered fixed effects, while experiments, replication within experiments, experiment by replication by genotype, and experiment by genotype were treated as random effects.

The height of each individual was measured at 3 dpi to identify the tall and short subsets and calculate the correlation between height and LEI (3, 7 and 14 dpi) and NTI (7 and 14 dpi). Also, correlation between LEI (7 and 14 dpi) and NTI (7 and 14 dpi) was calculated. Pearson's correlation coefficients were calculated using SAS Enterprise Guide 7.

Multiple observations of lesion size (mm) at 3, 7, and 14 dpi were used to calculate AUDPC in Excel for each genotype using the following formula, where y is disease level at time (t). The AUDPC is a quantitative measure of disease intensity with time (Simko and Piepho, 2012). A smaller value indicated a greater AUDPC.

$$\begin{array}{l} AUDPC=\overset{N_{i}-1}{\underset{i=1}{\sum }}\frac{\left(y_{i}+y_{i+1}\right)}{2}\left(t_{i+1}-t_{i}\right) \end{array}$$

Since LEI, LEIP, AUDPC, and SR were calculated from lesion size (mm) values, they are considered as LEI subsets in the QTL analysis.

Resistant genotypes to white mold were identified based on having low disease based on one or more of the three criteria. Those criteria include mean LEI 3 dpi equal or <25 mm, mean NTI after two weeks equal or greater than one, and survival rate of 25% or greater (Porter et al., 2009).

### Genotyping by Sequencing

DNA of PRIL17 and PRIL19 individuals were extracted using CTAB (Manfioletti and Schneider, 1988) and DArT (Diversity Arrays Technology, 2008) protocols. DNA of each individual was quantified using PicoGreen assay (Ahn et al., 1996) and restriction digested with *Ape*KI (Sonah et al., 2013). Ninety-six plex libraries of each population were constructed using the GBS protocol described by Elshire et al. (2011). GBS libraries of PRIL17 and PRIL19 were sequenced by single- end read sequencing using Illumina HiSeq 2000 at the McDermott Center (http://www.utsouthwesternedu/labs/dna-genotyping-core) and the Genomic Facility of Cornell University Biotechnology Resource Center (BRC), respectively. All sequencing datasets have been deposited with links to BioProject accession number PRJNA653945 in NCBI Sequence Read Archive (https://www.ncbi.nlm.nih.gov/bioproject/653945/).

### GBS Analysis and SNP Calling

Genotyping by sequencing raw data for PRIL17 and PRIL19 were analyzed using the GBS-SNP-CROP pipeline (Melo et al., 2016) by aligning the sequences of each population over the *Pisum_sativum*_v1a (Psat_v1a) reference genome (Kreplak et al., 2019). GBS-SNP-CROP pipeline is Perl scripts that implements parsing, filtering and SNP calling with bioinformatic tools such as Trimmomatic, and SAMtools. The GBS data was filtered with minimum coverage depth (minDP 3), maximum number of mismatches in alignment (*n* = 1), maximum missing data (max-missing 0.5), and minor allele frequency (maf 0.05). One hundred eighty-six individuals from each population and their parents were genotyped by GBS and analyzed using the GBS-SNP-CROP (GBS SNP-Calling Reference Optional) pipeline. Heterozygous and monomorphic parents were filtered out, and individuals with missing >60% SNPs were also excluded.

### Linkage Group Mapping

Linkage groups for each population were constructed using JoinMap® 4 (Plant Research International B.V and Kyazma B.V) (Van Ooijen, 2006). Linkage group formation was based on linkage LOD scores (logarithm of the odds). The linkage LOD score was calculated by JoinMap to compare the estimated value of the pairwise recombination frequency with 0.5. We used the start value of 3.0, end value of 20.0, and step size of 1.0 which indicated the ranges and steps of significance level that are used for grouping. After linkage groups were determined, the linkage map was constructed using the *Monte Carlo maximum likelihood* (ML) mapping algorithm for each group. Four physiological markers (*Le* for height*, er-1* for powdery mildew, *Pl* for hilum color, and *I* for cotyledon color) in PRIL17 and five physiological markers (*Le, er-1, Pl, I*, and *af* or afila) in PRIL19 were used to anchor linkage groups to study co-localization and association with disease resistance loci.

### Consensus Map Construction

A consensus map was created by QTL IciMapping V 4.2 software (Meng et al., 2015) from PRIL17 and PRIL19 linkage groups that shared common markers. The consensus map construction (CMP) function of QTL IciMapping has three steps to form an integrated map including grouping, ordering and rippling. All markers were assembled in one group and used the ordering algorithm of nearest neighbors with two-opt (nnTwoOp). The rippling criterion was SAD (Sum of Adjacent Distances), with the window size of five markers.

### QTL Mapping

QTL analysis was performed using linkage maps of PRIL17 and PRIL19, and least square means of all disease resistance components across experiments for each population. Data from greenhouse evaluations from each population were separated into tall (height > 150 mm) and short (height ≤ 150 mm) internode individuals and QTL analysis was conducted on each subset as well as the complete dataset. QTL analysis was conducted using the Inclusive Composite Interval Mapping (ICIM) method available in the software QTL IciMapping V 4.2 (Meng et al., 2015). Only unique loci from each linkage group were included in the analyses. Permutation tests based on 1,000 repeats were performed to determine significant LOD threshold values (α = 0.05 Type I error). Only the QTL detected above threshold LOD score were included in this study. If any such significant QTL was identified with LOD below the threshold, but > 2, the QTL were also included in the results as supporting information.

To compare the means of two group of RILs which differ for QTL alleles associated with a phenotypic trait, *t*-test (assuming unequal variances) function in Excel 2016 was used.

### Identification of Candidate Genes Associated With Resistance to White Mold

The QTL region associated with the flanking markers were used to search for candidate genes. Sequences of flanking markers were used to search the pea sequence database *Pisum sativum* v1a JBrowse (https://urgi.versailles.inra.fr/jbrowse/gmod_jbrowse/?data=myData/Pea/Psat_v1a/data) to find candidate genes related to each QTL. These markers in QTL region are associated with few genes and can be utilized as a first step for resistance breeding purposes in pea.

## Results

### Greenhouse Evaluation

Analysis of variance of checks across the experiments showed no significant difference (*P* > 0.05) (Table S1, Table S2), suggesting the data for different experiments could be combined for analysis for each population. Also, Levene's homogeneity test for LEI (3dpi) variance of checks for each population across different experiments showed no significant difference (PRIL17: *P* = 0.042, PRIL19: *P* = 0.0001), thus justifying a joining analysis across all experiments. Plant height showed a positive correlation with LEI and NTI (Table S3, Table S4). Survival rate of long internode individuals in both populations was noticeably higher than short internode individuals (Table S6). These may suggest that height might be a confounding factor and might obscure the real effect of *S. sclerotiorum* infection. Therefore, short and long internode individuals of each population were analyzed separately. A negative correlation coefficient was observed between LEI and NTI in the short and tall subsets of PRIL17 and PRIL19 (Table S5).

Genotypes with partial resistance were identified using the criteria of Porter et al. (2009). Analysis of the short internode subset of PRIL17 identified six individuals with LEI ≤ 25, thirty-three with SR ≥ 25% and 47 genotypes with NTI ≥ 1. The value of AUDPC ranged from 487 to 2270 in short genotypes of PRIL17. The AUDPC of PRIL17 parents, Lifter and PI240515, were 777 and 1119, respectively. Only PRIL17-181 among the short internode subset of PRIL17 showed all three of Porter's resistance criteria and low AUDPC (487). Seventeen short individuals of PRIL17 showed two of the resistant criteria together (Table 1).

**Table 1:** Lesion expansion inhibition (LEI) 3 days post inoculation (dpi), nodal transmission inhibition (NTI), and survival rate (SR) after 14 dpi and area under disease progress curve (AUDPC) from greenhouse evaluation and leaf type of the 22 short pea genotypes from PRIL17 and PRIL19 with greatest partial resistance to white mold.

**Short internode genotypes**	**LEI (3 dpi) mm**	**NTI (14 dpi)**	**SR**	**AUDPC**	**Leaf type**
**PRIL17**					
PRIL17-11	24	0.2	27	651	Normal
PRIL17-54	50	1.4	44	2,270	Normal
PRIL17-56	24	1.5	16	654	Normal
PRIL17-58	41	1.5	33	1,232	Normal
PRIL17-71	43	1.2	34	1,294	Normal
PRIL17-97	40	1.4	30	1,038	Normal
PRIL17-127	41	1.4	52	1,302	Normal
PRIL17-128	30	1.0	25	764	Normal
PRIL17-129	27	2.3	32	834	Normal
PRIL17-139	34	1.2	31	883	Normal
PRIL17-141	36	2.9	30	991	Normal
PRIL17-145	27	2.5	54	581	Normal
PRIL17-149	46	1.3	45	1,666	Normal
PRIL17-158	27	1.8	25	728	Normal
PRIL17-166	25	0.3	34	655	Normal
PRIL17-180	31	1.6	35	837	Normal
PRIL17-181	23	1.5	58	487	Normal
**PRIL19**					
PRIL19-18	63	1.4	34	2,336	Afila
PRIL19-74	74	2.3	53	2,401	Normal
PRIL19-86	38	2.8	36	1,822	Normal
PRIL19-124	37	1.2	35	1,027	Afila
PRIL19-127	29	1.4	35	930	Normal

Among long internode genotypes of PRIL17 only four genotypes restricted lesion expansion with low LEI (3 dpi). However, all long internode genotypes of PRIL17 showed NTI≥ 1 and 101 genotypes met the survival rate criteria. Only four tall genotypes (PRIL17-16, PRIL17-28, PRIL17-125, and PRIL17-179) from PRIL17 showed all three criteria together for partial resistance to white mold). PRIL17-125 showed all three of Porter's resistance criteria as well as low AUDPC (412). AUDPC for tall individuals of PRIL17 ranged from 412 to 3594.

Analysis of the short internode subset of PRIL19 identified one individual with LEI ≤ 25, five individuals with SR≥ 25% and five genotypes with NTI≥ 1. The AUDPC ranged from 824 to 3048 in short varieties of PRIL19. The AUDPC of PRIL19 parents, Medora and PI169603 were 1062 and 990, respectively. Although, none of the short individuals of PRIL19 met all three criteria, five of them met two criteria for white mold partial resistance (Table 1).

Analysis of the long internode subset of PRIL19 revealed that none of the individuals had LEI equal or <25 mm. Fifty-six of the tall subset of PRIL19 demonstrated NTI≥ 1 and forty-nine tall individuals with SR ≥ 25%. The AUDPC for tall individuals of PRIL19 ranged from 1107 to 3306.

Overall, twenty-two individuals from short genotypes of PRIL17 and PRIL19 showed partial resistance and met at least two of the resistance criteria in the greenhouse evaluation. Two of those RILs (PRIL19-18 and PRIL19-124) had afila leaf type as well (Table 1).

### Genetic Map Construction

For PRIL17, a total of 1985 polymorphic SNP markers were selected to construct the linkage map after filtering the genotypic data. Out of those markers, a total of 1967 SNPs were mapped onto seven linkage groups with a total map length of 1494 cM (Figure S1). The 1967 markers represented 899 unique loci with an average distance of 1.7 cM between two loci (Table 2). Height (*Le*), powdery mildew resistance (*er-1*), and hilum color (*Pl*) were anchored at 257-258 cM of chr5LG3, 65 cM of chr1LG6 and 170-171 cM chr1LG6, respectively, which aligned with the Weeden map (Bordat et al., 2011).

**Table 2:** Summary of genetic maps for PRIL17, PRIL19 and the consensus map.

**PRIL17 map**				**PRIL19 map**				**Consensus map**		
Chr*	**#Loci**	**#Unique loci**	**Map length cM**	**Chr**	**#Loci**	**#Unique loci**	**Map length cM**	**Chr**	**#Unique loci**	**Map length cM**
chr1LG6	209	114	173.8	chr1LG6	118	70	203.8	chr1LG6	275	250.6
chr2LG1	211	77	159.2	chr2LG1(a)	80	56	93.8	chr2LG1	150	247.1
ch3LG5	292	129	182.0	chr2LG1(b)	50	30	35.9	ch3LG5	206	353.8
ch4LG4	314	156	288.9	ch3LG5	178	109	203.8	ch4LG4	242	483.5
ch5LG3	323	152	271.4	ch4LG4	170	114	215.7	ch5LG3	206	353.8
ch6LG2	257	110	213.8	ch5LG3(a)	263	149	297.2	ch6LG2	160	336.3
ch7LG7	361	161	204.9	ch5LG3(b)	14	7	6.0	ch7LG7	247	436.2
				ch6LG2	145	72	165.7			
				ch7LG7	179	117	192.9			
Total	1967	899	1494	Total	1197	724	1415	Total	1486	2461

* *Pea chromosome number, chr refers to chromosome number of pea genome assembly v1a and LG refers to linkage group of earlier genetic mapping studies (Tayeh et al., 2015)*.

For PRIL19, a total of 1,207 polymorphic SNP markers were selected after filtering the genotypic data. Out of those, a total of 1196 markers were mapped to nine linkage groups with a total map distance of 1415 cM (Figure S2). The 1196 markers represented 724 unique loci with an average distance of 1.9 cM between loci (Table 2). Height (*Le*), powdery mildew resistance (*er-1*), leaf type (*af* ) and hilum color (*Pl*) were anchored at 296-297 cM of chr5LG3(a), 60 cM of chr1LG6, 8–16 cM of chr2LG1(b), and 129–130 cM of chr1LG6, respectively, which aligned with the Weeden map (Bordat et al., 2011). Cotyledon color (*I*) was positioned at 95 cM of chr5LG3(a).

### Consensus Map Construction

A total of 1486 SNPs were assembled into seven linkage groups. These markers spanned over 2461 cM with an average distance of 1.7 cM between two markers (Figure S3, Table 2).

### Quantitative Trait Loci Mapping

#### PRIL17 (Lifter × PI240515)

**Figure 1:**
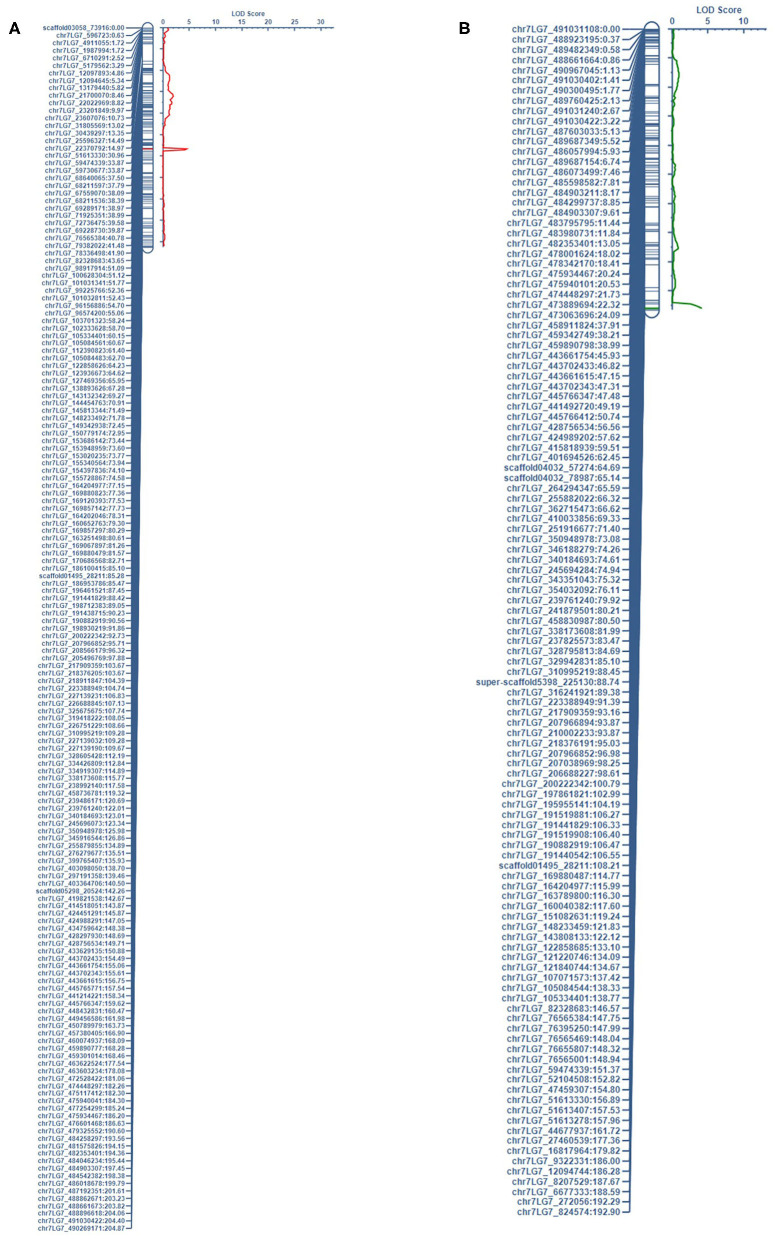
The QTL associated with LEI on chr7LG7 was detected in the short internode subset of **(A)** PRIL17 (*QLEI.17.ndsu.7*) and **(B)** PRIL19 (*QLEI.19.ndsu.7*) populations.

**Figure 2:**
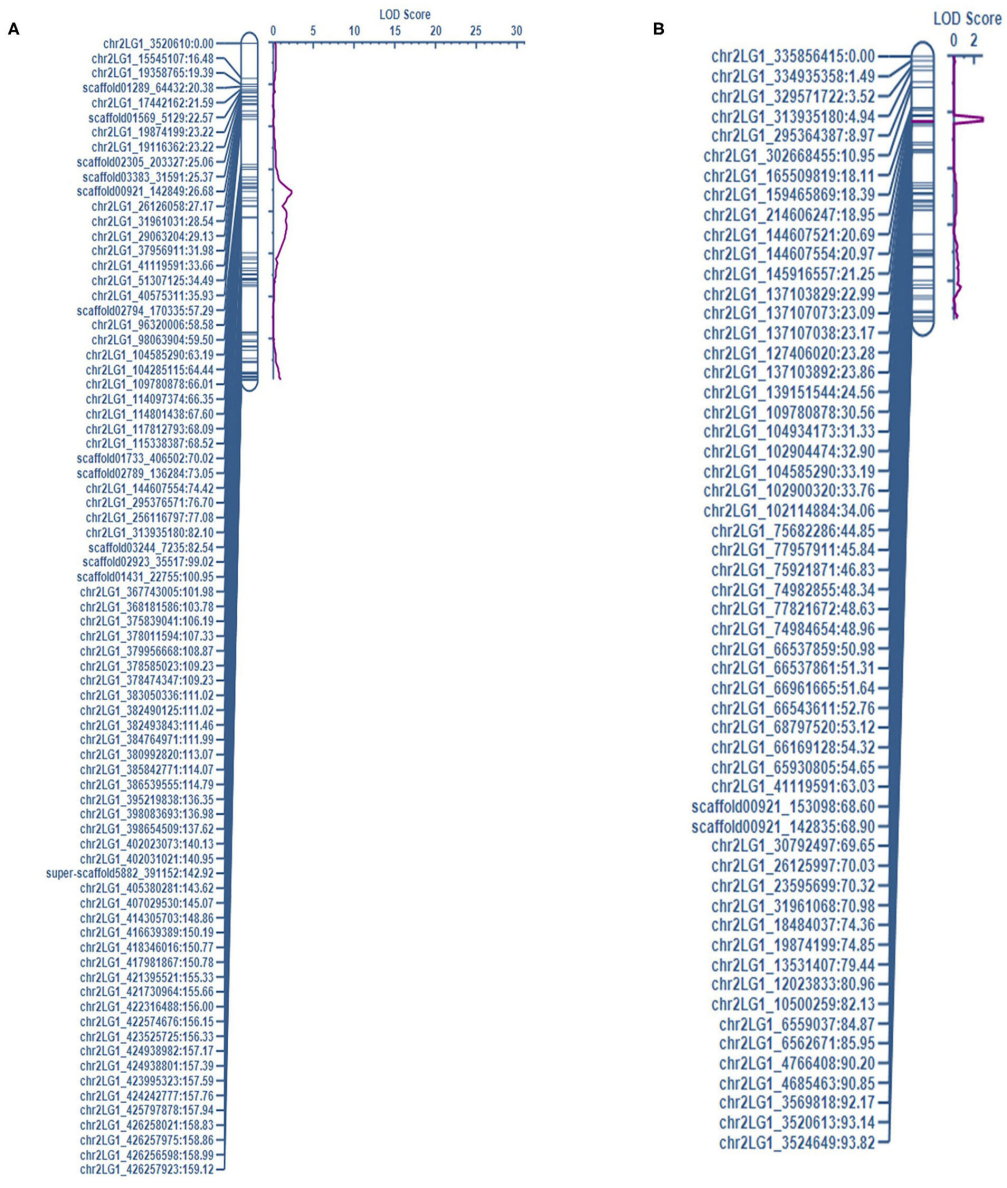
The QTL associated with NTI on chr2LG1 were detected in the complete dataset of **(A)** PRIL17 (*QNTI.17.ndsu.2*) and, **(B)** PRIL19 (*QNTI.19.ndsu.2*) populations.

This study identified five QTL for LEI, three QTL for NTI, three for AUDPC, one for SR, and one for LEIP in PRIL17 (Table 3). There were some common QTL between traits which means the total of seven unique QTL were identified in PRIL17 population. A significant QTL associated with LEI, NTI, LEIP, and SR was identified at 255–264 cM on chr5LG3 in all three datasets. This QTL was located at the same position as height (*Le*) gene (Weeden et al., 1998; Lee et al., 2017) (LOD = 22.8 and r^2^ = 45.8%). The genotype Lifter contributed the alleles for increased value of LEI, and PI240515 alleles contributed to increased values of NTI, LEIP, and SR at all the identified loci (**Table 5**). Common QTL associated with LEI (*QLEI.17.ndsu.6*) and AUDPC (*QAUDPC.17.ndsu.6*) was identified at 88 cM of chr6LG2 in a short internode subset with LOD = 3.1, where Lifter contributed the allele for improved value of traits (**Table 5**). Another common QTL for LEI (*QLEI.17.ndsu.2*) and AUDPC (*QAUDPC.17.ndsu.2*) was identified at 157-158 cM of chr2LG1 in short internode individuals subset with the LOD = 3.7 and the allele for increased value of traits were conferred by Lifter. One more QTL for LEI was identified at 85-114 cM of chr7LG7 (*QLEI.17.ndsu.7*, LOD = 4.7) in complete datasets as well as short internode individuals' subset (Figure 1A). The separation of RILs based on parental alleles at this locus showed that the non-adapted PI240515 parent, improved LEI (**Table 5**). Also, one minor QTL for LEI (*QLEI.17.ndsu.4*), and two minor QTL for NTI (*QNTI.17.ndsu.2* and *QNTI.17.ndsu.1*) were identified in the complete dataset of PRIL17 (Figure 2A, Table 3).

**Table 3:** QTL for reaction to white mold infection identified in PRIL17 based on all individuals, short internode, and long internode genotype subsets.

**QTL and trait**	**Dpi^a^**	**Chr^b^**	**Position (cM)**	**Other associated traits**	**Dataset type^c^**	**Left marker**	**Right marker**	**LOD**	**R^2^^e^**	**Add^f^**
**HEIGHT**										
Le**		chr5LG3	246-265	LEI, NTI, AUDPC SR, LEIP	All, short, tall	chr5LG3_557023279^d^	chr5LG3_569105948	25.5	48.7	100.7
**LEI (cm)**										
QLEI.17.ndsu.2*	7	chr2LG1	157-158	AUDPC	Short	chr2LG1_425797878	chr2LG1_426258021	3.7	12.4	−154.3
*QLEI.17.ndsu.4*	3	chr4LG4	42		All	chr4LG4_25831962	scaffold01160_24125	2.8	5.1	−2.5
QLEI.17.ndsu.5**	3,7,14	chr5LG3	255-264	*Le*, NTI, SR, LEIP, AUDPC	All, short	chr5LG3_562563492	chr5LG3_568430003	22.8	45.8	46.9
QLEI.17.ndsu.6***	7	chr6LG2	88	AUDPC	Short	chr6LG2_163685348	chr6LG2_159992168	3.0	11.2	9.3
*QLEI.17.ndsu.7*	3,7	chr7LG7	85-114		All, short	chr7LG7_170686568	chr7LG7_334919307	4.7	8.9	3.3
**NTI**										
*QNTI.17.ndsu.1*	14	chr1LG6	0		All	chr1LG6_27014318	chr1LG6_27820673	2.3	3.4	0.2
*QNTI.17.ndsu.2*	14	chr2LG1	70		All	chr2LG1_115338387	scaffold01733_406502	2.3	3.5	−0.19
QNTI.17.ndsu.5**	7,14	chr5LG3	254-260	*Le*, LEI, SR, LEIP	All, tall	chr5LG3_562563492	chr5LG3_570275258	16.3	32.2	0.6
**AUDPC**										
QAUDPC.17.ndsu.2*		chr2LG1	157	LEI	All	chr2LG1_423525725	chr2LG1_424938982	3.7	12.4	−154.3
QAUDPC.17.ndsu.5**		chr5LG3	257-260	*Le*, NTI, LEI, SR, LEIP	Short	chr5LG3_566156376	chr5LG3_570275258	3.0	11.1	171.0
QAUDPC.17.ndsu.6***		chr6LG2	88	LEI	Short	chr6LG2_163685348	chr6LG2_159992168	3.1	12.1	151.9
**SR**										
QSR.17.ndsu.5**		chr5LG3	261-262	*Le*, LEI, NTI, LEIP	All	chr5LG3_570780892	chr5LG3_569106574	14.9	31.0	−9.9
**LEIP**										
QLEIP.17.ndsu.5**		chr5LG3	255-260	*Le*, LEI, NTI, SR	All	chr5LG3_562563492	chr5LG3_570275258	14.6	31.5	9.9

a Number of days relative QTL was found post inoculation of the lesion expansion inhibition or nodal transmission inhibition traits.

b Pea chromosome number, chr refers to chromosome number of pea genome assembly v1a and LG refers to linkage group of earlier genetic mapping studies (Kreplak et al., 2019).

c Type of datasets where relative QTL was found. all: All the genotypes in dataset were used for QTL analysis; short: short subset of the dataset was used for QTL analysis; tall: tall subsets of dataset were used for QTL analysis.

d In each SNP ID, chr refers to chromosome number of pea genome assembly v1a and LG refers to linkage group of earlier genetic mapping studies followed by the base pair position. For non-chromosomal SNPs, scaffold followed by the scaffold number and base pair position (Kreplak et al., 2019).

e Phenotypic variation explained by QTL.

f Additive effect.

*, **, ***: QTL with the same markings are the same.

Few RILs were identified with a combination of positive alleles at the identified QTL. PRIL17 line120 carried the alleles for increased trait value for *QLEI.17.ndsu.2, QNTI.17.ndsu.2, QLEI.17.ndsu.4, QLEI.17.ndsu.6*, and *QLEI.17.ndsu.7* loci (AA+ BB+ AA+ AA+ BB). The alleles for increased trait value for *QNTI.17.ndsu.2, QLEI.17.ndsu.4, QLEI.17.ndsu.6, QLEI.17.ndsu.7* and *QLEI.17.ndsu.5* loci (BB+ AA+ AA+ BB+ AA) were carried by PRIL17-83, PRIL17-157, PRIL17-164 and PRIL17-166. The desirable allele for *QLEI.17.ndsu.2, QNTI.17.ndsu.2, QLEI.17.ndsu.4, QLEI.17.ndsu.5*, and *QLEI.17.ndsu.7* and loci (AA+ BB+ AA+ AA+ BB) were carried by PRIL17-141. The PRIL17-145 also had positive allele to increase trait value for *QNTI.17.ndsu.2, QLEI.17.ndsu.6*, and *QLEI.17.ndsu.7* loci (BB+ AA+ BB).

#### PRIL19 (PI169603 × Medora)

In population PRIL19, CIM identified four QTL for LEI, two each for NTI and AUDPC, and one each for SR and LEIP (Table 4). Some of the QTL were common among different traits and six unique QTL associated with white mold resistance in PRIL19. Also, two QTL for height were identified in PRIL19; one of them being located at the position of the Mendel internode length (*Le*) gene.

**Table 4:** QTL for reaction to white mold infection identified in PRIL19 based on all individuals, short internode, and long internode genotype subsets.

**QTL and trait**	**Dpi^a^**	**Chr^b^**	**Position (cM)**	**Other associated traits**	**Dataset type^c^**	**Left marker**	**Right marker**	**LOD**	**R^2^^e^**	**Add^f^**
**HEIGHT**										
Le.ndsu*		chr5LG3(a)	0-5	LEI, AUDPC	All, short	chr5LG3_569106574^d^	chr5LG3_561561067	21.5	61.5	83.3
*Le*.**		chr5LG3(a)	296	NTI, LEIP, SR	All	chr5LG3_566442038	chr5LG3_566847213	23.6	64.3	0.4
**LEI (cm)**										
QLEI.19.ndsu.5.1*	3,7,14	chr5LG3(a)	0-5	*Le.ndsu*, AUDPC	All	chr5LG3_569106574	chr5LG3_561561067	9.3	34.1	37.7
QLEI.19.ndsu5.2***	7	chr5LG3(a)	150-152	AUDPC	Short	chr5LG3_139674536	chr5LG3_138696064	3.2	22.4	−7.5
*QLEI.19.ndsu.4*	3	chr4LG4	174-184		Short	chr4LG4_52663612	scaffold01646_102677	2.3	19	4.6
*QLEI.19.ndsu.7*	7	chr7LG7	191-192		Short	chr7LG7_6677333	chr7LG7_272056	4.2	27.4	−8.3
**NTI**										
*QNTI.19.ndsu.2*	14	chr2LG1(a)	22-45		All, tall	chr2LG1_145916557	chr2LG1_77957911	2.8	13.0	0.3
QNTI.19.ndsu.5**	7,14	chr5LG3(a)	296-297	*Le*, LEIP, SR	All, tall	chr5LG3_568430003	chr5LG3_569648908	20.3	50.4	0.7
**AUDPC**										
QAUDPC.19.ndsu.5.1***		chr5LG3(a)	167	LEI	Short	chr5LG3_148081339	chr5LG3_167547321	2.7	21.8	−122.4
QAUDPC.19.ndsu.5.2*		chr5LG3(a)	0-6	*Le.ndsu*	All	chr5LG3_569106574	chr5LG3_561561067	16.1	45.7	410.2
**SR**										
QSR.19.ndsu.5**		chr5LG3(a)	297	*Le*, NTI, LEIP	All	chr5LG3_568430003	chr5LG3_569648908	14.7	42.6	−11.2
**LEIP**										
QLEIP.17.ndsu.5**		chr5LG3(a)	297	*Le*, NTI, SR	All	chr5LG3_568430003	chr5LG3_569648908	14.0	40.9	11.1

a Number of days relative QTL was found post inoculation of the lesion expansion inhibition or nodal transmission inhibition traits.

b Pea chromosome number, chr refers to chromosome number of pea genome assembly v1a and LG refers to linkage group of earlier genetic mapping studies (Kreplak et al., 2019).

c Type of datasets where relative QTL was found. all: All the genotypes in dataset were used for QTL analysis; short: short subset of the dataset was used for QTL analysis; tall: tall subsets of dataset were used for QTL analysis.

d In each SNP ID, chr refers to chromosome number of pea genome assembly v1a and LG refers to linkage group of earlier genetic mapping studies followed by the base pair position. For non-chromosomal SNPs, scaffold followed by the scaffold number and base pair position (Kreplak et al., 2019).

e Phenotypic variation explained by QTL.

f Additive effect.

*, **, ***: QTL with the same markings are the same.

The most significant QTL identified at 296 cM on chr5LG3(a), was associated with NTI, LEIP and SR in the complete data set as well as the long internode subset. This QTL was located at the same position as the height (*Le*) gene with LOD= 20.4 which explained up to 50.4% of the phenotypic variance (Weeden et al., 1998; Lee et al., 2017). The PI169603 alleles contributed to increased value of NTI, LEIP and SR at all the identified loci (Table 5). Another significant common QTL between LEI and AUDPC was identified at 0-6 cM on chr5LG3(a) in the complete datasets. This QTL was located at the same position as the height QTL (*Le.ndsu*) and explained 45.7% of the phenotypic variation. Interestingly, non-adapted parent, PI169603 conferred alleles for increased height at *Le.ndsu* (Table 5).

**Table 5:** Parental alleles related to increased and decreased trait values and observed significant mean differences in the individuals^*^ for phenotypic trait values when a specific parental allele exists at QTL identified in this study.

**QTL**	**Related trait**	**Allele for increased trait value**	**Trait value**	**Allele for decreased trait value**	**Trait value**	***T*-test for mean difference (*P* value)**
**PRIL17**						
*Le*	Height	BB	358.2	AA	125.6	1.12916E-27
*QNTI.17.ndsu.1*	NTI(14 dpi)	AA	2.5	BB	1.9	0.002264
*QLEI.17.ndsu.2*	LEI(7 dpi)	AA	52.6	BB	74.9	0.003389
*QNTI.17.ndsu.2*	NTI(14 dpi)	BB	2.4	AA	1.9	0.014825
*QLEI.17.ndsu.4*	LEI(3 dpi)	AA	36.1	BB	41.3	0.002767
*QLEI.17.ndsu.5*	LEI(3 dpi)	AA	34.6	BB	42.7	2.27648E-06
	LEI(7 dpi)	AA	62.3	BB	112.3	2.63E-15
	LEI(14 dpi)	AA	91.3	BB	176.7	7.32092E-14
	NTI(7 dpi)	BB	3.3	AA	2.4	1.95E-12
	NTI(14 dpi)	BB	2.8	AA	1.4	5.98E-16
	LEI%	BB	54.2	AA	75	5.69E-11
	SR%	BB	46.1	AA	27.2	3.43442E-10
	AUDPC	AA	1049.8	BB	1912.5	1.42E-19
*QLEI.17.ndsu.6*	LEI(7 dpi)	AA	55.5	BB	72.8	0.0171
	AUDPC	AA	946.4	BB	1217.6	0.019231
*QLEI.17.ndsu.7*	LEI(3 dpi)	BB	37.5	AA	43.3	0.002568
	LEI(7 dpi)	BB	84.2	AA	99.4	0.028224
*QLEI.17.ndsu.4+ QLEI.17.ndsu.7*	LEI(3 dpi)	AA+ BB	33.1	BB+ AA	46.3	0.001287
*QLEI.17.ndsu.2+ QLEI.17.ndsu.6*	LEI(3 dpi)	AA+ AA	48	BB+ BB	86.5	0.003701
	AUDPC	AA+ AA	792.7	BB+ BB	1435.3	0.002521
**PRIL19**						
*Le*	Height	AA	275.3	BB	148.6	1.37315E-06
*Le.ndsu*	Height	AA	298.1	BB	133.7	1.66032E-21
*QNTI.19.ndsu.2*	NTI(14 dpi)	AA	1.8	BB	1.1	0.031602
*QLEI.19.ndsu.4*	LEI(3 dpi)	BB	37.4	AA	43.8	0.053682
*QLEI.19.ndsu.5.1*	LEI(3 dpi)	BB	44.6	AA	58.1	1.53E-05
	LEI(7 dpi)	BB	86.5	AA	127	6.35E-08
	LEI(14 dpi)	BB	120.8	AA	193.5	2.76E-10
	AUDPC	BB	1390.3	AA	2176.2	2.04E-14
*QLEI.19.ndsu.5.2*	LEI(7 dpi)	AA	72.3	BB	83.9	0.004942
	AUDPC	AA	1215.9	BB	1419.7	0.002687
*QNTI.19.ndsu.5*	NTI(14 dpi)	AA	1.7	BB	0.3	4.57393E-24
	LEI%	AA	71.1	BB	94.2	2.26713E-18
	SR%	AA	28.6	BB	5.8	1.24479E-17
*QLEI.19.ndsu.7*	LEI(7 dpi)	AA	69.6	BB	87.8	0.002838

* Only the individuals showing a clear genotype for all the associated markers at the target QTL were used for the analysis. *AA = Lifter allele; BB = PI240515 allele (PRIL17)*. *AA = PI169603 allele; BB = Medora allele (PRIL19)*.

For LEI, a QTL was identified in the short internode subset at 148-190 cM on chr5LG3(a) (*QLEI.19.ndsu5.2*) and explained 22.4% of phenotypic variation. The desirable allele for improving LEI were contributed through PI169603 (Table 5). Another QTL for LEI was found in short internode subset at 191–192 cM of chr7LG7 (*QLEI.19.ndsu.7*) (Figure 1B). This QTL explained 27.4% of the phenotypic variation with LOD = 4.2 which PI169603 provided alleles for improving LEI at all the identified loci. Also, one minor QTL for LEI (*QLEI.19.ndsu.4*) and one minor QTL for NTI (*QNTI.19.ndsu.2*) were identified in different datasets of PRIL19 (Figure 2B).

The PRIL19-16, PRIL19-101, PRIL19-124, PRIL19-127, PRIL19-135 possessed the desirable alleles for improving trait value for *QLEI.19.ndsu.5.1, QLEI.19.ndsu.5.2*, and *QLEI.19.ndsu.7* loci (BB+ AA+ AA). Also, the positive alleles for increased traits value for *QLEI.19.ndsu.5.1, QLEI.19.ndsu.5.2, QLEI.19.ndsu.7*, and *QLEI.19.ndsu.4* loci (BB+ AA+ AA+ BB) were carried by PRIL19-104.

#### Identification of Candidate Genes Associated With Resistance to White Mold

Genome browsing of flanking marker positions of QTL associated with white mold resistance with the *Pisum sativum* sequence revealed forty genes involved in plant defense mechanisms (Table 6).

**Table 6:** Genes found from genome browsing of flanking markers associated with resistance to white mold in *Pisum sativum* v1a JBrowse (Kreplak et al., 2019).

**QTL**	**SNP**	**Overlapping genes**	**Gene ID^b^*Pisum sativum* v1a**	**GO ID^c^**
**PRIL17**				
*QNTI.17.ndsu.1*	chr1LG6_27014318^a^	Leucine rich repeats (2 copies)	Psat1g019120	0030275
	chr1LG6_27820673	Amino acid permease	Psat1g019680	0015171
*QLEI.17.ndsu.2*	chr2LG1_423525725	PH domain profile	Psat2g185280	0042731
	chr2LG1_424938982	KH domain	Psat2g187240	
	chr2LG1_425797878	Protein kinase domain profile	Psat2g189080	
	chr2LG1_426258021	DYW family of nucleic acid deaminases	Psat2g189520	
*QNTI.17.ndsu.2*	chr2LG1_115338387	Ethylene-responsive protein kinase Le-CTR1	Psat2g065120	
	scaffold02789_136284	Metal-dependent phosphohydrolase+ HD subdomain	Psat0s2789g0080	
*QLEI.17.ndsu.4*	chr4LG4_25831962	Ligase activity+ forming carbon-nitrogen bonds	Psat4g018040	0016879
	scaffold01160_24125	IQ calmodulin-binding motif	Psat0s1160g0040	0005516
*QLEI.17.ndsu.5*	chr5LG3_566156376	Hsp70 protein	Psat5g299000	0030544
	chr5LG3_570275258	C2 domain	Psat5g302040	
	chr5LG3_554216613	MULE transposase domain	Psat5g288200	
	chr5LG3_562563492	Taurine catabolism dioxygenase TauD+ TfdA family	Psat5g295640	
	chr5LG3_566847213	Cyclin+ N-terminal domain	Psat5g299560	
	chr5LG3_570780892	Hpt domain	Psat5g302360	0009927
	chr5LG3_569106574	Ribosomal protein S11	Psat5g300640	0003735
	chr5LG3_557023279	Rab-GTPase-TBC domain	Psat5g290800	0017137
	chr5LG3_579082448	Tub family	Psat5g308240	
*QLEI.17.ndsu.6*	chr6LG2_163685348	Zinc finger+ C3HC4 type (RING finger)	Psat6g099280	0071535
*QLEI.17.ndsu.7*	chr7LG7_334919307	UEV domain	Psat7g177600	
	chr7LG7_334426809	Enoyl-(Acyl carrier protein) reductase	Psat7g177080	0016631
	chr7LG7_186100415	Clathrin light chain	Psat7g112920	0071439
	chr7LG7_170686568	Reverse transcriptase-like	Psat7g105080	
**PRIL19**				
*QNTI.19.ndsu.2*	chr2LG1_75682286	Seven in absentia protein family	Psat2g042720	
	chr2LG1_77957911	Squalene/phytoene synthase	Psat2g044520	0051996
	chr2LG1_102114884	Ankyrin repeats (3 copies)	Psat2g058000	0005515
	chr2LG1_137103829	Cellular nucleobase+ nucleoside+ nucleotide and nucleic acid metabolic process	Psat2g073040	0006139
*QLEI.19.ndsu.4*	scaffold01646_102677	Protein of unknown function (DUF789)	Psat0s1646g0040	
*QLEI.19.ndsu.5.1*	chr5LG3_569648908	Polypeptide deformylase	Psat5g301200	0042586
	chr5LG3_568430003	Ion transport protein	Psat5g077720	0006811
	chr5LG3_566442038	ESCO1/2 acetyl-transferase	Psat5g299360	0007062
	chr5LG3_566847213	Cyclin+ N-terminal domain	Psat5g299560	
*QLEI.19.ndsu5.2*	chr5LG3_99955442	Hydrolase activity+ acting on acid anhydrides+ catalyzing transmembrane movement of substances	Psat5g055560	0016817
	chr5LG3_148081339	F-box-like	Psat5g082440	
	chr5LG3_167547321	GDP-fucose protein O-fucosyltransferase	Psat5g091800	0008417
*QNTI.19.ndsu.5*	chr5LG3_561561067	Helix-loop-helix DNA-binding domain	Psat5g294600	
*QLEI.19.ndsu.7*	chr7LG7_272056	Rhodanase C-terminal	Psat7g000320	
	chr7LG7_6677333	Elongation factor Tu GTP binding domain	Psat7g004080	

a In each SNP ID, chr refers to chromosome number of pea genome assembly v1a and LG refers to linkage group of earlier genetic mapping studies (Tayeh et al., 2015) followed by the base pair position. For non-chromosomal SNPs, scaffold followed by the scaffold number and base pair position.

b Gene IDs were provided by (Kreplak et al., 2019).

c *Gene Ontology (GO) ID refers to the statement about the molecular function of a particular gene (Binns et al., 2009)*.

## Discussion

Breeding white mold resistant pea varieties is challenging due to several reasons. There is limited insight into genes related to the pathogen-host interaction (pathogenicity and resistance genes) due to the absence of high levels of resistance in pea germplasm. The *S. sclerotiorum* pathogen infects a broad host range including important crops such as soybean, bean, canola, and sunflower, with different mechanisms and there is no report of complete resistance in any crop (Bolton et al., 2006). Therefore, in this study, we characterized two pea populations to identify partial resistance genotypes and dissect the genetics associated with white mold resistance.

Analyzing a detached stem assay to identify potential sources of white mold resistance in pea plants and comparision with the a whole plant assay in a previous study (Ashtari Mahini, 2018) indicated that pathogen might behave differently in detached stems and the result might be associated with senescence other than defense pathways. We observed negative phenotypic correlation between LEI and NTI similar to Porter et al. (2009). This suggests different mechanisms are operating in the resistant reaction as reported in GWAS (genome-wide association study) analysis and RNA sequencing of the pea-*S. sclerotiorum* interaction (Chang et al., 2018). The RNA sequence analysis of Lifter and PI240515 at 12, 24, and 48 h post inoculation showed that more leucine rich-repeat containing transcripts and oxidoreductase transcripts were associated with greater lesion resistance, while VQ (Valine-glutamine) motif-containing proteins and a myo-inositol oxygenase increased for nodal resistance (Chang et al., 2018).

A small number of pea genotypes restricted lesion advancement in this study, but most of them did not survive after 14 days. This indicates that NTI should be combined with LEI to prevent lesion development through the stem (Porter et al., 2009). The lower AUDPC values indicate slower disease development and greater resistance to the disease compared to larger AUDPC values (Kull et al., 2004). However, this was not always true in our experiment.

Although, there is no direct relationship between stem strength and disease resistance to white mold (Porter, 2012; Ashtari Mahini, 2018), incorporating physical characters such as stem thickness, short internode and the afila leaf type can help develop genetically resistant genotypes which are commercially acceptable (Porter, 2012).

The inheritance of resistance to white mold is partial and quantitative as demonstrated by the distribution of phenotypic data for disease resistance and several observed putative QTL in this study. It seems that physiological resistance and morphological avoidance are the components of the partial resistance of pea to white mold. Plant height is most likely associated with disease escape. In soybean varieties, response to *S. sclerotiorum* also depends on disease escape due to height, maturity and reduced lodging (Boland and Hall, 1986). Overall, it may be better and more reasonable to select QTL for physiological resistance mechanisms which contribute to restrict the development of the pathogen in pea.

Although, there have been some reports of QTL identification for *S. sclerotiorum* resistance in sunflower (Micic et al., 2005), soybean (Bastien et al., 2014; Iquira et al., 2015), bean (Kolkman and Kelly, 2003; Ender and Kelly, 2005; Miklas, 2007), rapeseed (*Brassica napus*) (Wu et al., 2013), and *Brassica oleracea* (Mei et al., 2013), there is only one such report in pea (Tashtemirov, 2012). Until now, the only QTL mapping study for resistance to *S. sclerotiorum* in pea was conducted using F_2_ lines from the cross between Lifter and PI240515. Two QTL (linked to SSR markers AA255 and AD73) were found on LG2 and LG3 related to NTI and LEI, respectively (Tashtemirov, 2012). We verified QTL associated with LEI on chr5LG3 in all three data type sets of each of the populations. Compared to earlier report (Tashtemirov, 2012), we observed more QTL in our study. Higher number of QTL identified in this study could be attributed to better experimental design and higher number of recombination events in RILs compared to F_2_ families.

There were thirteen QTL identified which three of them were in the same loci as height gene (*Le*) and the other ten QTL associated with two forms of white mold resistance. Seven QTL contributed in reducing the rate of lesion development with LEI (LEIP, AUDPC, and SR considered as LEI subsets). Three QTL restricted development of the pathogen through the node (NTI) in the pea stem. The QTL associated with LEI on chr7LG7 was detected in the short internode subset of both populations (Figure 1). Likewise, the QTL associated with NTI on chr2LG1 were detected in the complete dataset of both populations (Figure 2).

The sequence analysis of the flanking markers of QTL associated with white mold resistance identified candidate genes that may be involved in resistance. We observed different candidate resistance genes for LEI and NTI. Candidate genes such as protein kinase, elongation factors, ion transport, C2 domain, heat shock protein 70, F box protein, and RING (Really Interesting New Gene) zinc finger were related to LEI, and genes such as ethylene-responsive protein, helix-loop-helix DNA-binding domain, ankyrin repeats, and leucine rich repeats (LRR) were associated to NTI. This further indicates the possibility of different genetic mechanisms underlying LEI and NTI as has been observed earlier (Chang et al., 2018). However, in contrast to the observations by Chang et al. (2018), we found that the LRR protein related to NTI and protein kinase was associated with LEI in our study.

The sequence analyses found that our QTL correspond to some candidate genes that have earlier been reported in transcriptome or proteomics analysis of various hosts during *S. sclerotiorum* infection. For example, *EF-Tu* (elongation factor thermo unstable) plays important roles in providing stress adaptation and is down regulated in the proteomics analysis of pea plants infected with *S. sclerotiorum* (Jain et al., 2015). The ion transport proteins provide a channel for Ca^2+^ flow across membrane and enable Ca^2+^ elevation in response to pathogen signals (Wang et al., 2019). On the other hand, C2 domain of *B. napus* interacts with the polygalacturonases (PGs) effector of *S. sclerotiorum* and involves in calcium signaling processes during plant infection (Wang et al., 2009). Ethylene-responsive protein and KH domain are involved in hormonal response ethylene and jasmonate pathway, respectively (Zhao et al., 2007; Thatcher et al., 2015). Protein kinase domain and genes encoding transcription factors such as RING zinc finger protein, and helix-loop-helix DNA-binding domain play a part in signal pathway (Zhao et al., 2007). Leucine rich repeats protein contribute to R-gene based resistance and differentially expressed during *S. sclerotiorum* and *B. napus* interaction (Zhao et al., 2007; Wei et al., 2016; Wu et al., 2016). Heat shock protein 70 (Hsp70), F box like protein, and ankyrin repeat involve in abiotic stress, protein degradation and metabolism, respectively and were induced during *S. sclerotiorum* invasion in *B. napus* (Zhao et al., 2007, 2009).

For pre-breeding purposes, we were able to find some partial resistance RILs with a combination of desirable alleles for important loci from both adapted and non-adapted parents. These lines include PRIL17-141, PRIL17-145, PRIL17-166, PRIL19-124, and PRIL19-127. These germplasm and markers developed in this study will facilitate marker-assisted selection for white mold resistant cultivars in pea.

## Conclusion

In this study, two RIL populations were developed to study the genetics of white mold resistance in pea. Extensive phenotyping combined with genotyping by sequencing, identified a total of ten QTL associated with white mold resistance. Seven of those QTL were associated with LEI and three QTL were associated with NTI. About 50% of the beneficial QTL alleles for increased LEI and NTI were contributed by adapted parents in both populations which indicate the transgressive segregation due to combination of superior alleles. The non-native parents also contributed important loci for LEI and NTI and provide the opportunity to improve the adapted gene pool for white mold resistance. This further proved the importance of wild germplasm to broaden the gene pool and introgress lost beneficial genetic diversity to cultivated germplasm.

Twenty-two inbred lines with short plant stature were identified showing partial resistance to white mold and met at least two of the resistance criteria in the greenhouse evaluation as proposed by Porter et al. (2009). The partial resistant RILs having combinations of valuable alleles from both parents at identified loci could be important material for breeding programs to enhance resistance traits. To conclude, the pre-breeding material, genomic and genetic resources developed in this study could be exploited through marker-assisted breeding programs for developing white mold resistant cultivars in pea.

## Data Availability Statement

The original contributions presented in the study are included in the article/Supplementary Materials, further inquiries can be directed to the corresponding author/s.

## Author Contributions

RA conceptualized and carried out the experiments on the material that KM and LP provided for the project. RA analyzed and wrote the manuscript with support from AK and JF who helped to analyse the data. KM and EE helped supervise and conceptualize the project. AK, JF, LP, EE, and KM also helped to review and edited the manuscript. All authors contributed to the article and approved the submitted version.

## Conflict of Interest

The authors declare that the research was conducted in the absence of any commercial or financial relationships that could be construed as a potential conflict of interest. The reviewer KG declared a past collaboration with one of the authors KM to the handling editor.
