# Supplementary material for: Analysis and Identification of QTL for Resistance to Sclerotinia sclerotiorum in Pea (Pisum sativum L.)
Source: Front Genet. 2020 Nov 19;11:587968. doi: 10.3389/fgene.2020.587968 (PMC7710873; doi:10.3389/fgene.2020.587968)
Supplement: Supplementary file 1 [file Table_1.docx]

Table ‎S1: ANOVA of LEI (mm) at 3 days post inoculation (dpi) for five check genotypes (Lifter, PI240515, Stirling, Bohatyr and Shawnee) tested with PRIL17 across three experimental runs.

| Source | DF | Sum of Squares | Mean Square | F-Value | Pr > F |
| --- | --- | --- | --- | --- | --- |
| Rep | 3 | 567.4 | 189.2 | 1.29 | 0.36 ^ns^ |
| Exp | 2 | 204.4 | 102.2 | 0.38 | 0.70 ^ns^ |
| Genotype | 3 | 382.0 | 127.3 | 0.52 | 0.70 ^ns^ |
| Rep (Exp) | 6 | 881.3 | 146.9 | 1.26 | 0.30 ^ns^ |
| Exp* Genotype | 6 | 1478.8 | 246.5 | 2.12 | 0.07 ^ns^ |
| Error | 37 | 4302.8 | 116.3 |  |  |

^ns^ not significant.

Table S2: ANOVA of LEI (mm) at 3 days post inoculation for five check genotypes (Medora, PI169603 Stirling, Bohatyr and Shawnee) tested with PRIL19 across three experimental runs.

| Source | DF | Sum of Squares | Mean Square | F-Value | Pr > F |
| --- | --- | --- | --- | --- | --- |
| Rep | 3 | 693.5 | 231.2 | 1.81 | 0.25 ^ns^ |
| Exp | 2 | 41.1 | 20.6 | 0.20 | 0.83 ^ns^ |
| Genotype | 3 | 2247.2 | 749.1 | 9.05 | 0.01**** |
| Rep (Exp) | 6 | 765.8 | 127.6 | 1.20 | 0.33 ^ns^ |
| Exp* Genotype | 6 | 496.6 | 82.8 | 0.78 | 0.60 ^ns^ |
| Error | 37 | 3924.4 | 106.1 |  |  |

^ns^ not significant.

** p< 0.01.

Table ‎S3: Pearson’s correlation coefficient for correlations between LEI, NTI and plant height (3dpi) for PRIL17. *dpi: days post inoculation. N= 2268

|  | LEI (3 dpi*) | LEI (7 dpi) | NTI (7 dpi) | LEI (14 dpi) | NTI (14 dpi) |
| --- | --- | --- | --- | --- | --- |
| Height | 0.28**** | 0.56**** | 0.48**** | 0.75**** | 0.60**** |

** p< 0.01

Table S4: Pearson’s correlation coefficient for correlations between LEI, NTI and plant height (3dpi) for PRIL19. *dpi: days post inoculation. N= 1531.

|  | LEI (3 dpi*) | LEI (7 dpi) | | NTI (7 dpi) | LEI (14 dpi) | NTI (14 dpi) |
| --- | --- | --- | --- | --- | --- | --- |
| Height | 0.45**** | 0.60**** | 0.55**** | | 0.72** | 0.61**** |

** p< 0.01

Table S5: Pearson’s correlation coefficient and p-value for correlations between LEI, NTI data in short and tall subset of PRIL17 and PRIL19.

|  | PRIL17 | | | | | | | |  |  | PRIL19 | | | | | | |
| --- | --- | --- | --- | --- | --- | --- | --- | --- | --- | --- | --- | --- | --- | --- | --- | --- | --- |
|  |  | LEI (7dpi^a^) | |  | |  | LEI (14dpi) | |  |  | LEI (7dpi) | |  |  | LEI (14dpi) | |  |
|  | Short | | Tall | | Short | | | Tall | | Short | | Tall | | Short | | Tall | |
| NTI (7 dpi) | -0.09**** | | -0.24** | |  | | |  | | -0.14****** | | -0.41*** | |  | |  | |
| NTI (14 dpi) |  | |  | | -0.18****** | | | -0.30** | |  | |  | | -0.23****** | | -0.40*** | |

^a^ day post inoculation.

** p< 0.01

*** p< 0.001

**** p< 0.0001

Table S6: Descriptive statistics of survival rate (SR) of short and long internode subsets of PRIL17 and PRIL19.

|  |  | PRIL17 | |  |  | PRIL19 |  |
| --- | --- | --- | --- | --- | --- | --- | --- |
| Test statistic | Short | | Tall | | Short | | Tall |
| Mean | 21.7 | | 45.6 | | 6.17 | | 29.2 |
| Std. Dev. | 24.9 | | 19.8 | | 14.7 | | 21.7 |
| Std. Error | 0.8 | | 0.6 | | 0.6 | | 0.8 |
| Variance | 620.8 | | 390.9 | | 216.5 | | 472.5 |
| Minimum | 0.0 | | 0.0 | | 0.0 | | 0.0 |
| Maximum | 96 | | 96.9 | | 88.1 | | 95.7 |
