# Supplementary material for: Analysis and Identification of QTL for Resistance to Sclerotinia sclerotiorum in Pea (Pisum sativum L.)
Source: Front Genet. 2020 Nov 19;11:587968. doi: 10.3389/fgene.2020.587968 (PMC7710873; doi:10.3389/fgene.2020.587968)
Supplement: Supplementary file 2 [file Table_2.docx]

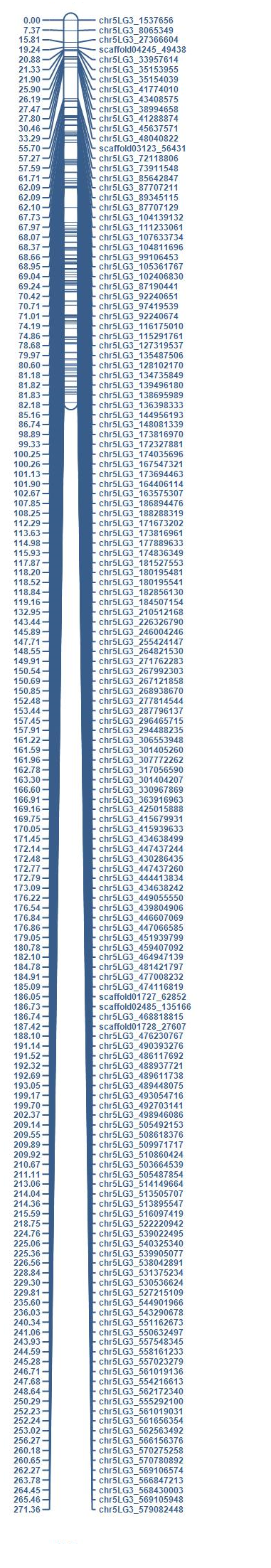

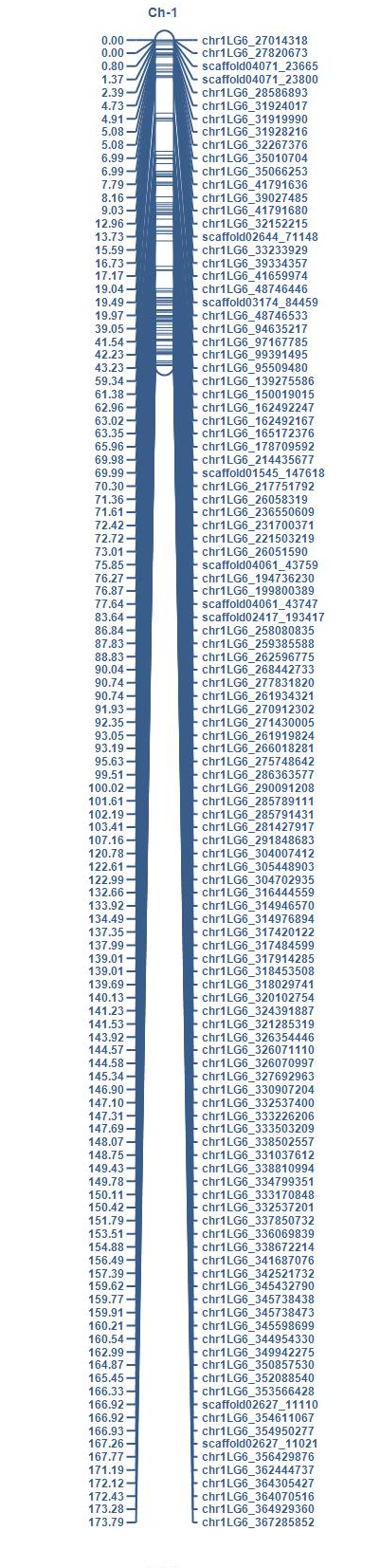

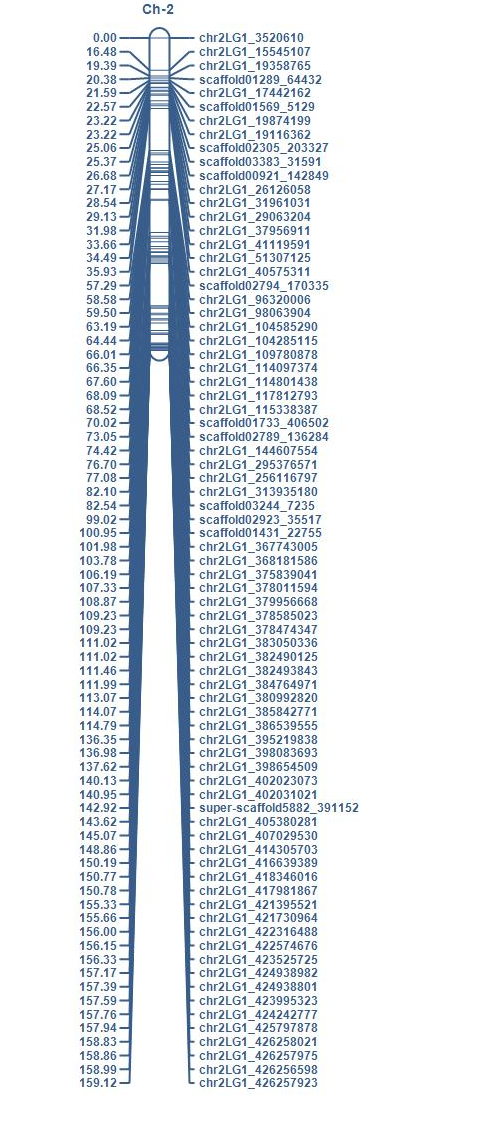

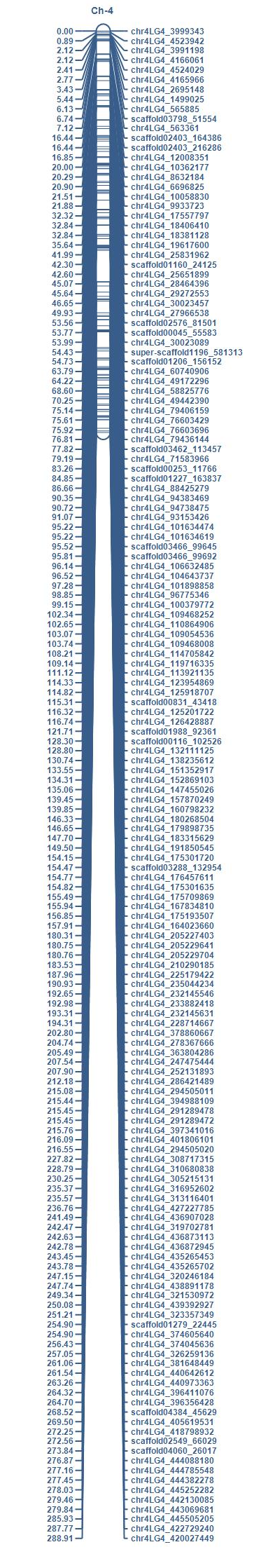

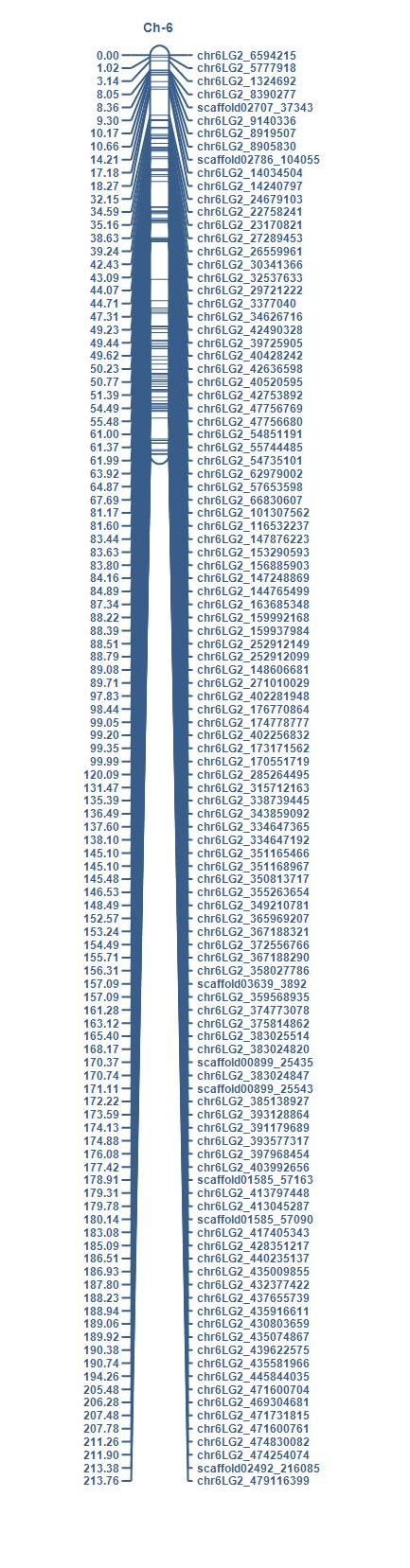

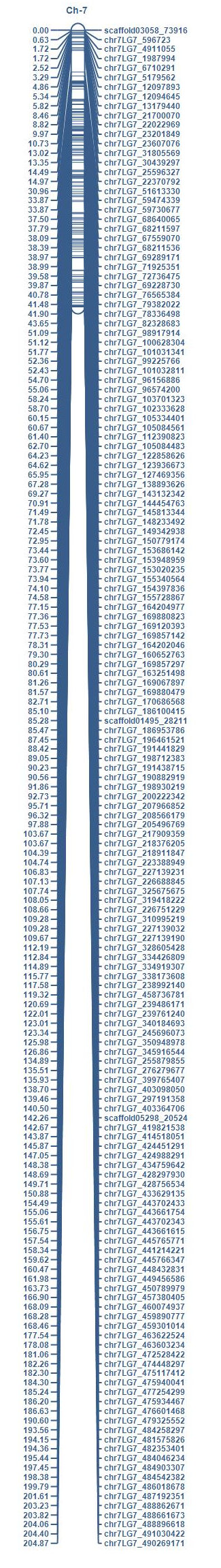

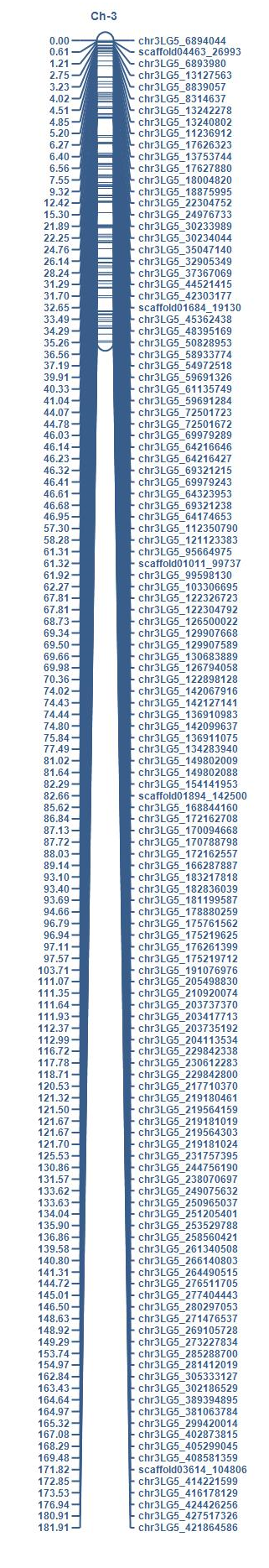


chr1LG6

chr2LG1

chr3LG5

chr5LG3

chr6LG2

chr7LG7

chr4LG4

*Le, QLEI.17.ndsu.5 QNTI.17.ndsu.5*

*QLEI.17.ndsu.2*

*QLEI.17.ndsu.4*

*QLEI.17.ndsu.6*

*QLEI.17.ndsu.7*

*QNTI.17.ndsu.1*

*QNTI.17.ndsu.2*

Figure ‎S1: Linkage map for PRIL17 based on SNPs derived from genotype by sequencing and corresponding QTL associated with white mold resistance.
