# Supplementary material for: Analysis and Identification of QTL for Resistance to Sclerotinia sclerotiorum in Pea (Pisum sativum L.)
Source: Front Genet. 2020 Nov 19;11:587968. doi: 10.3389/fgene.2020.587968 (PMC7710873; doi:10.3389/fgene.2020.587968)
Supplement: Supplementary file 3 [file Table_3.docx]

Figure S2: Linkage map for PRIL19 based on SNPs derived from genotype by sequencing and corresponding QTL associated with white mold resistance.


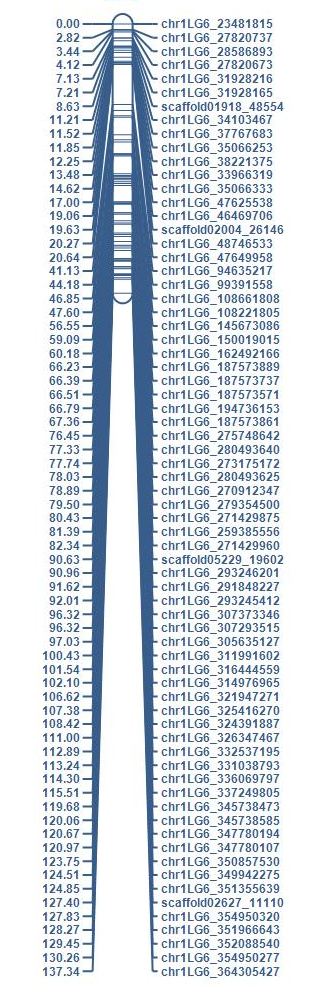

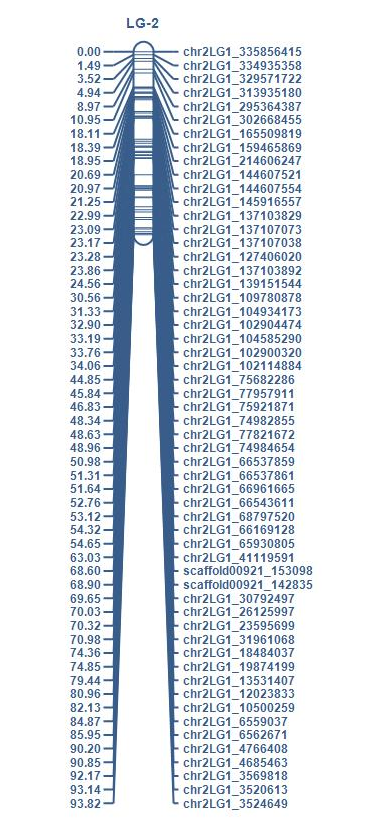

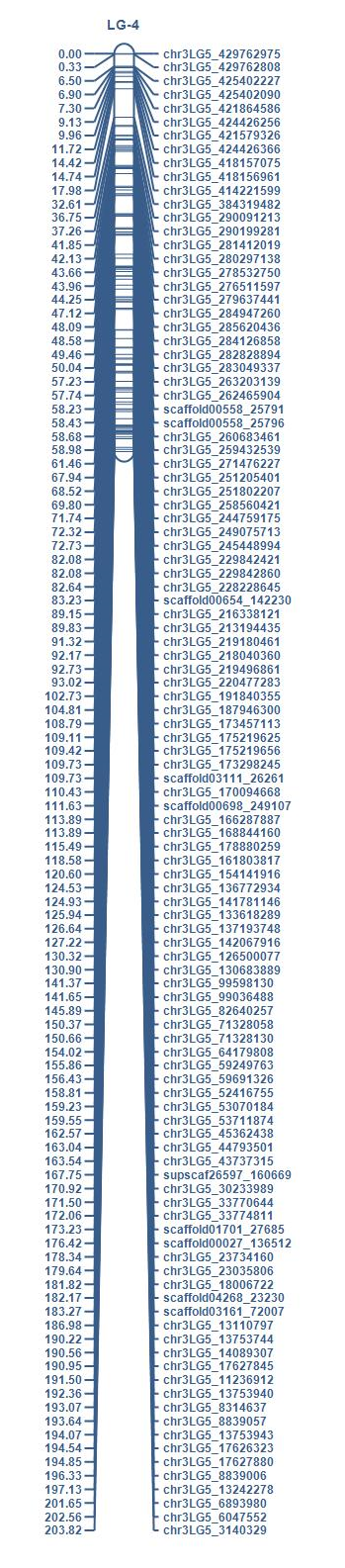

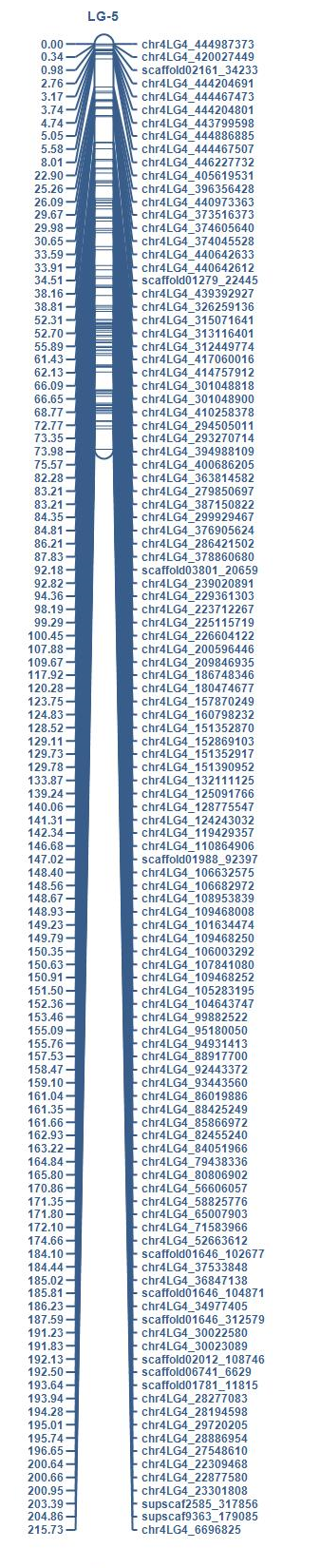

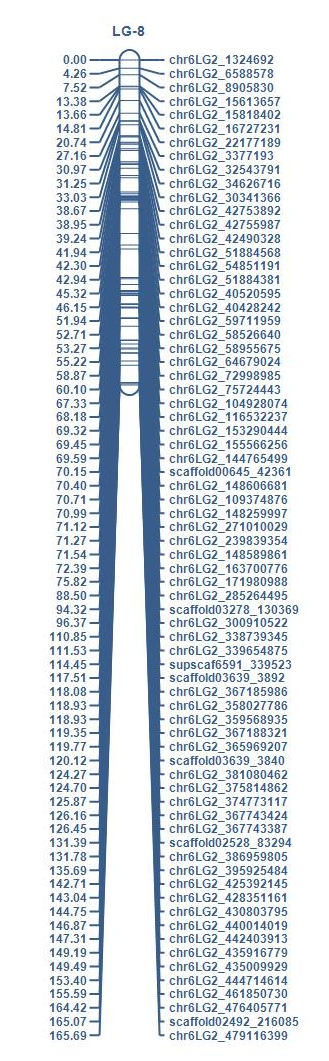

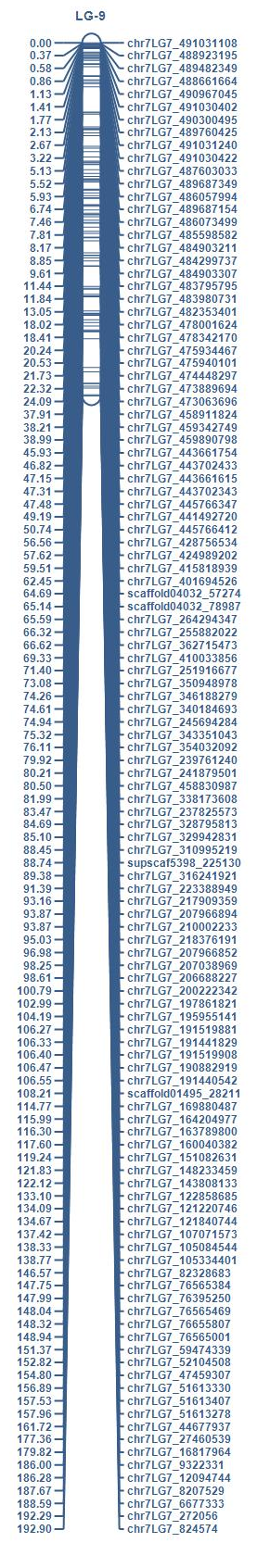


chr2LG1(a)

chr1LG6

chr2LG1(b)

chr3LG5

chr4LG4

chr5LG3(a)

chr5LG3(b)

chr6LG2

chr7LG7


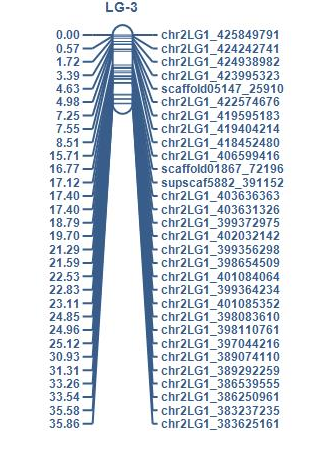

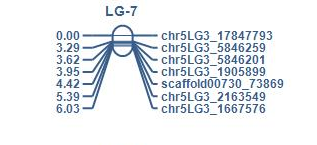

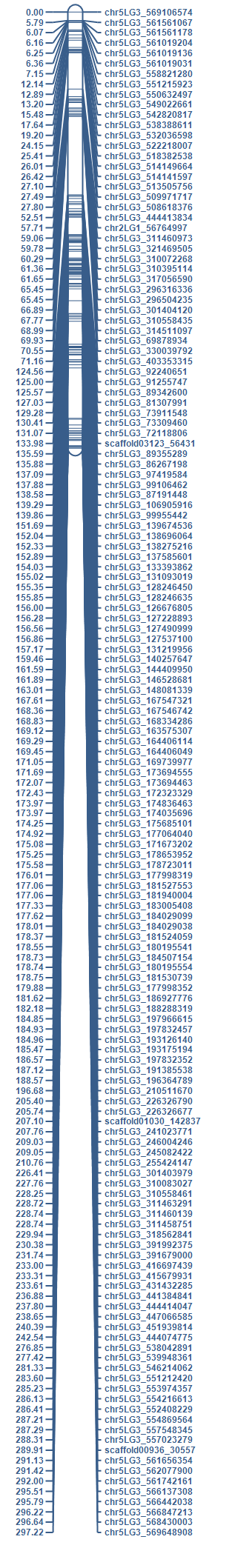


*Le,* *QNTI.19.ndsu.5, QLEIP.17.ndsu.5*

*Le.ndsu, QLEI.19.ndsu.5.1*

*QLEI.19.ndsu5.2*

*QLEI.19.ndsu.7*

*QLEI.19.ndsu.4*

*QNTI.19.ndsu.2*
