# Supplementary material for: Analysis and Identification of QTL for Resistance to Sclerotinia sclerotiorum in Pea (Pisum sativum L.)
Source: Front Genet. 2020 Nov 19;11:587968. doi: 10.3389/fgene.2020.587968 (PMC7710873; doi:10.3389/fgene.2020.587968)
Supplement: Supplementary file 4 [file Table_4.docx]

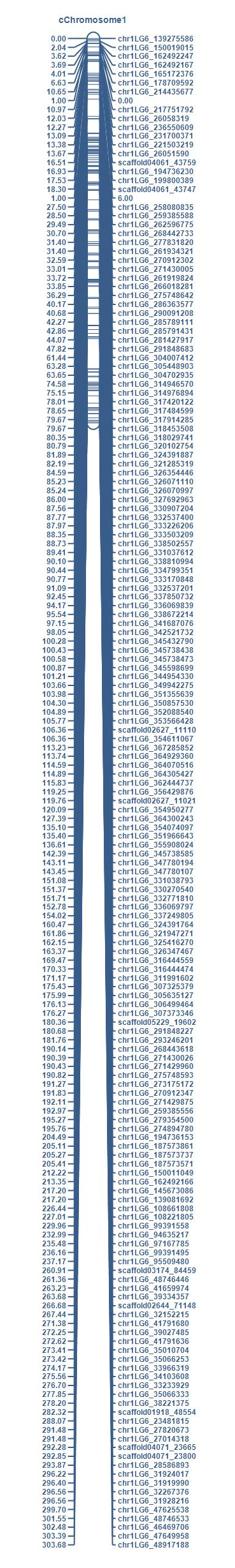

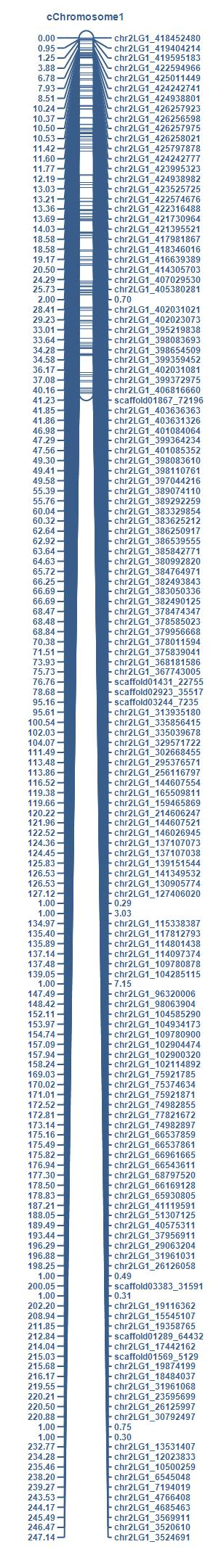

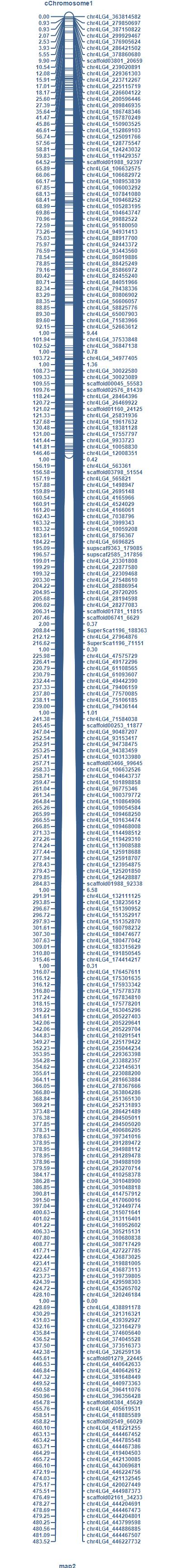

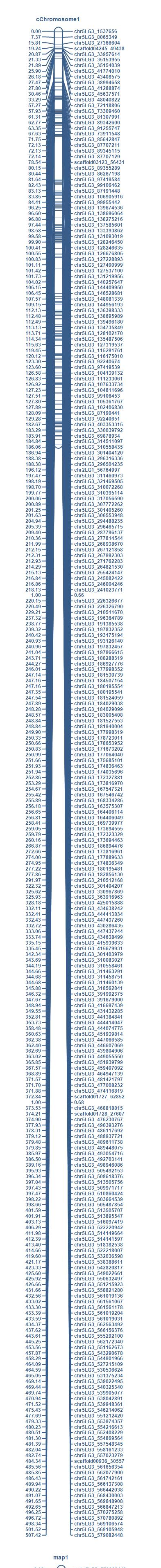

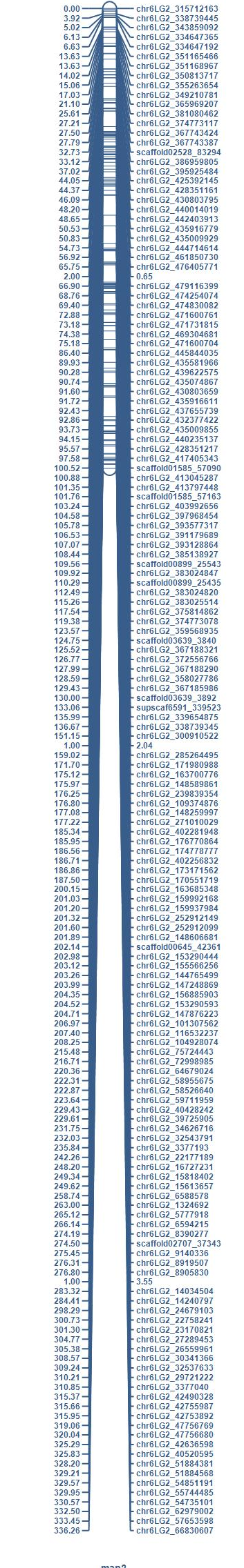

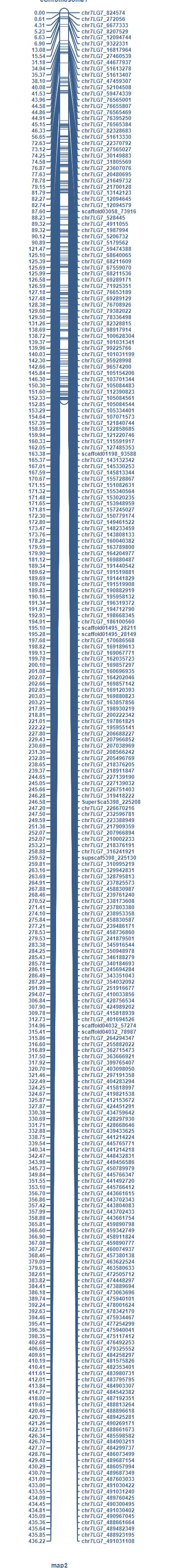


chr1LG6

chr2LG1

chr3LG5

chr4LG4

chr5LG3

chr6LG2

chr7LG7


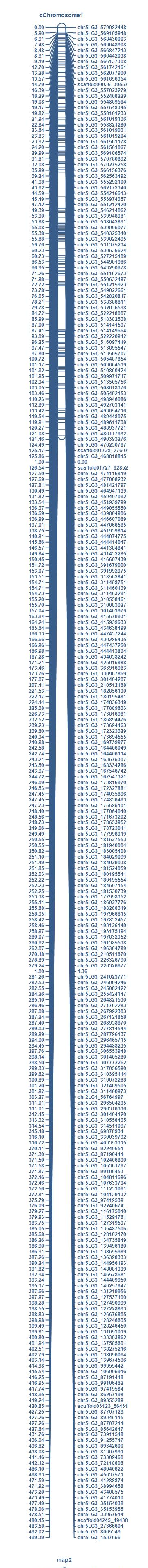


Figure S3: Composite map generated from PRIL17 and PRIL19 linkage groups that were sharing the common markers.
